# Understanding the Genetic Landscape of Gestational Diabetes: Insights into the Causes and Consequences of Elevated Glucose Levels in Pregnancy

**DOI:** 10.3390/metabo14090508

**Published:** 2024-09-20

**Authors:** Caroline Brito Nunes, Maria Carolina Borges, Rachel M. Freathy, Deborah A. Lawlor, Elisabeth Qvigstad, David M. Evans, Gunn-Helen Moen

**Affiliations:** 1 Institute for Molecular Bioscience, The University of Queensland, Brisbane 4067, Australia; 2 MRC Integrative Epidemiology Unit, University of Bristol, Bristol BS8 1QU, UK; 3 Population Health Sciences, Bristol Medical School, University of Bristol, Bristol BS8 2PS, UK; 4 Department of Clinical and Biomedical Sciences, Faculty of Health and Life Sciences, University of Exeter, Exeter EX4 4PY, UK; r.freathy@exeter.ac.uk; 5 Institute of Clinical Medicine, Faculty of Medicine, University of Oslo, 0372 Oslo, Norway; 6 Department of Endocrinology, Morbid Obesity and Preventive Medicine, Oslo University Hospital, 0424 Oslo, Norway; 7 Frazer Institute, University of Queensland, Brisbane 4102, Australia; 8 K.G. Jebsen Center for Genetic Epidemiology, Department of Public Health and Nursing, NTNU, Norwegian University of Science and Technology, 7491 Trondheim, Norway

**Keywords:** gestational diabetes, glucose metabolism, pregnancy, diagnosis, genetics

## Abstract

**Background/Objectives:** During pregnancy, physiological changes in maternal circulating glucose levels and its metabolism are essential to meet maternal and fetal energy demands. Major changes in glucose metabolism occur throughout pregnancy and consist of higher insulin resistance and a compensatory increase in insulin secretion to maintain glucose homeostasis. For some women, this change is insufficient to maintain normoglycemia, leading to gestational diabetes mellitus (GDM), a condition characterized by maternal glucose intolerance and hyperglycaemia first diagnosed during the second or third trimester of pregnancy. GDM is diagnosed in approximately 14.0% of pregnancies globally, and it is often associated with short- and long-term adverse health outcomes in both mothers and offspring. Although recent studies have highlighted the role of genetic determinants in the development of GDM, research in this area is still lacking, hindering the development of prevention and treatment strategies. **Methods:** In this paper, we review recent advances in the understanding of genetic determinants of GDM and glycaemic traits during pregnancy. **Results/Conclusions:** Our review highlights the need for further collaborative efforts as well as larger and more diverse genotyped pregnancy cohorts to deepen our understanding of the genetic aetiology of GDM, address research gaps, and further improve diagnostic and treatment strategies.

## 1. Pathophysiology of GDM

**Figure 1:**
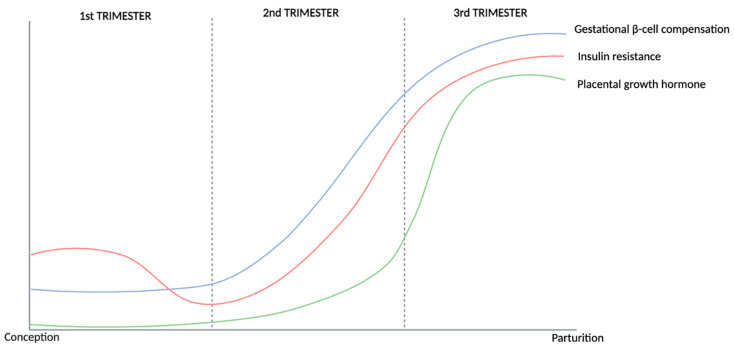
Illustration depicting key changes in glucose metabolism that occur during a healthy pregnancy based on the literature reviewed and summarised above. Throughout pregnancy, the body undergoes a dynamic adjustment to maternal insulin resistance through β-cell compensation. This shift in insulin resistance is influenced by placental growth hormone, which acts locally to induce insulin resistance in maternal peripheral tissues. To counterbalance this, gestational β-cell compensation begins in the second trimester, marked by increased insulin secretion, and reaches its peak level in the third trimester, ensuring adequate glucose regulation despite increased insulin resistance. GDM, however, might occur if glucose utilization and the compensatory increase in insulin secretion are not sufficient to reduce and maintain blood glucose levels within the regulated range. GDM: gestational diabetes mellitus.

In a healthy pregnancy, metabolic changes are essential to meet the energy expenditure required to support the growing fetus, with maternal glucose metabolism in particular providing necessary nutrition for healthy fetal growth. Changes in maternal glucose metabolism during pregnancy are characterized by elevated postprandial blood glucose levels, increased insulin resistance, and a compensatory increase in the secretion of insulin (Figure 1) [1,2,3,4,5,6,7,8,9,10,11,12]. These adaptations are essential to prepare the mother’s body for the metabolic demands of fetal growth, which includes transfer of nutrients from the mother to the developing fetus, as well as the provision of additional energy storage for both lactation and delivery [12].

Fasting glucose (FG) levels decrease in the first trimester of pregnancy, likely as a result of increased plasma volume induced by early hormone changes, with these values stabilizing in the second trimester largely due to fetal utilization, before further decreasing during the third trimester [11,13]. An increased resistance to the action of insulin (compared with pre-pregnancy levels) is also observed in healthy pregnancies, resulting in higher postprandial glucose levels during gestation [1,6,7,11,14,15,16]. This insulin resistance increases as the pregnancy progresses through the second and third trimesters due to the influence of placental hormones (prolactin, human placental lactogen, and human placental growth hormone) and pro-inflammatory cytokines [1,2,3,4,5,11,17,18,19,20,21,22,23,24]. Additionally, this state of insulin resistance enhances endogenous glucose production and breakdown of stored fat, which consequently leads to a further increase in blood glucose and free fatty acid levels [23].

Although insulin resistance is a common and essential physiological change during pregnancy, gestational diabetes mellitus (GDM) manifests when the concomitant compensatory increase in insulin secretion that occurs during pregnancy does not counterbalance insulin resistance and, consequently, is unable to maintain glucose homeostasis [5]. This mechanism, known as β-cell compensation, is characterized by β-cell mass expansion and other key molecular changes that are necessary to increase insulin secretion and maintain normoglycemia [24,25,26,27,28,29,30]. As such, the inability of β-cells to produce more insulin can lead to maternal hyperglycaemia and increased levels of glucose crossing the placenta, causing the fetus to produce excess insulin, a known fetal growth factor, which can lead to excessive fetal growth and associated perinatal complications [30,31,32,33,34].

## 2. GDM Diagnosis and Screening

For GDM diagnosis, blood glucose levels during pregnancy are commonly measured as overnight FG as well as one- and two-hour (and/or more rarely three-hour) glucose plasma values post oral glucose tolerance test (OGTT). This test is typically conducted at 24–28 weeks of gestation, a period historically observed to show the greatest variation in glucose levels [35]. For pregnant individuals undergoing an OGTT, blood samples are first taken after an overnight fast to measure fasting glucose levels. Then, additional blood samples are collected at one, two, and/or three hours after the ingestion of a 75 g glucose solution to further assess glucose levels. Although all these measurements can be used to diagnose hyperglycaemia, higher FG levels are observed in response to insulin resistance whilst post-load OGTT plasma glucose values have been reported to better reflect glucose uptake in skeletal muscles and disturbances in insulin production and secretion (indicative of β-cell function), respectively [36,37,38].

The screening strategies and diagnostic criteria employed to identify GDM cases vary substantially across regions. These differences include variations in gestational age at screening, whether screening is targeted solely at high-risk women or universally applied as well as plasma cut-off levels used during the OGTT (for an overview of the variations in GDM diagnostic criteria, see Table 1). Despite ongoing research and discussions within the medical community, a consensus has yet to be reached regarding the optimal diagnostic and screening criteria for identifying mothers with GDM. As can be seen in Table 1, not only GDM screening approaches but also GDM diagnostic criteria vary considerably across regions and have changed over time. The most recent international criteria, developed by the International Association of Diabetes in Pregnancy Study Group (IADPSG) in 2010 and also supported by the World Health Organization (WHO), recommend a universal 75 g OGTT screening between 24 and 28 weeks of gestation. This recommendation is based on the Hyperglycemia and Adverse Pregnancy Outcome (HAPO) study findings, comprised of 23,316 women of multiple ethnic and geographic origins, which reported clear linear associations between maternal plasma glucose levels after a 75 g OGTT and a variety of maternal and fetal adverse outcomes such as large for gestational age, neonatal hypoglycaemia, and frequency of Caesarean sections [39,40,41,42]. The IADPSG committee suggested that prespecified odds of adverse outcomes should be used to set the thresholds of glucose levels for defining GDM cases based on the average glucose values at which odds for birth weight > 90th percentile, cord C-peptide > 90th percentile, and percent body fat > 90th percentile reached 1.75 times the estimated odds of these outcomes at mean glucose values (values based on adjusted logistic regression models) [39]. 

It is important to emphasize that although aiming to improve the diagnosis of GDM and find the best approach to reduce adverse outcomes (given existing resources and budgets), the thresholds set by IADPSG are arbitrary as there is no threshold effect (in the HAPO results). Consequently, this has led to criticism with regards to the implementation of a universal GDM screening using the IADPSG criteria as this approach could lead to the misdiagnosis of women with moderate hyperglycaemia and unnecessary prescription of medications in what was previously considered healthy pregnancies. Although seen to reduce the risk of offspring large for gestational age, preterm birth, and neonatal hypoglycaemia overall, this lower diagnostic threshold has also been shown to provide limited benefits to women without additional risk factors [43,44]. 

## 3. Epidemiology of GDM

**Figure 2:**
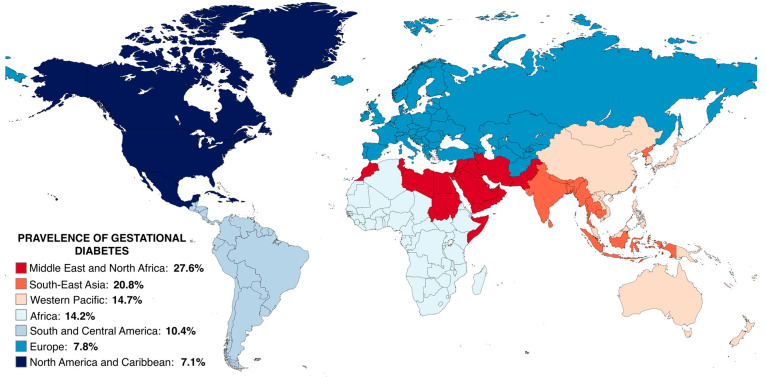
Estimated prevalence of gestational diabetes across global regions; prevalences taken from Wang et al., 2022 [45] and visualised using MapChart. A fixed-effects meta-analysis of 57 studies covering 45 countries was performed, with the diagnostic criteria and universal OGTT strategy proposed by IADPSG, as well as the age group of 25–30 years, serving as benchmarks for standardizing the prevalence of GDM across various practices and age groups. The global standardized prevalence was reported to be 14.2%, with the pooled prevalence in pregnant women around 25–30 years of age being 27.6%, 20.8%, 14.7%, 14.2%, 10.4%, 7.8%, and 7.1% in the Middle East and North Africa (MENA), South-East Asia (SEA), Western Pacific (WP), Africa (AFR), South and Central America (SACA), Europe (EUR), and North America and Caribbean (NAC). Although the study controlled for diagnostic criteria, screening approach, and age group, population characteristics were not taken into account. Pooled prevalence should be interpreted with caution as it was calculated based on varying and arbitrary cut points. OGTT: Oral glucose tolerance test; IADPSG: International Association of the Diabetes and Pregnancy Study Group; GDM: gestational diabetes mellitus.

GDM presents a significant challenge to maternal health, being diagnosed in approximately 14.0% of all pregnancies or roughly one in six births worldwide [45], though these figures are importantly influenced by marked variation in screening (i.e., universal vs. risk-based) and the thresholds used to diagnose GDM. Globally, the reported prevalence of GDM varies widely, with Middle Eastern and North African countries having the highest prevalence (27.6%) and North America and Caribbean regions having the lowest (7.1%) (Figure 2) [45]. Understanding the global burden of GDM has been challenging for decades due to the lack of uniform screening strategies and diagnostic criteria for GDM, variations in the prevalence of diagnosed type-I and II diabetes (as women with pre-existing diabetes follow a different obstetric care-path and are not screened or tested for GDM), and the diversity in antenatal health care practices across regions.

A systematic review and meta-analysis including up to 136,705 women was performed to compare the GDM prevalence after implementing the new IADPSG criteria with the GDM prevalence when older GDM criteria were used [46]. This study reported a 75% increase in the number of women diagnosed with GDM, with the overall effect estimates showing high heterogeneity in the pooled analysis. Subgroup analyses were undertaken for maternal age, BMI, study design, screening method, early screening, and use of modified IADPSG criteria, with a possible suggestion of such differences; however, there was no exploration of these differences statistically. 

Whilst the adoption of a lower diagnostic threshold and recommendation of a universal screening have contributed to an increase in the number of identified GDM cases, an upward trend in GDM prevalence was already evident prior to the implementation of these new criteria [47,48,49,50,51,52]. Over the past few decades, for instance, the demographic profile of pregnant women has changed significantly, with women having children at a more advanced age in high- and some middle-income countries, and obesity rising globally, both of which are likely to have further contributed to an increase in the prevalence of GDM [47,53,54]. 

## 4. Aetiology of GDM

GDM has multiple genetic, lifestyle, and clinical risk factors contributing to disease onset and progression. However, these risk factors have been mostly investigated in traditional observational epidemiological studies, which are often prone to confounding by social, environmental, and behavioural factors. Genetics, however, provides an opportunity to inform on potential causal relationships between traditional risk factors and disease through the application of the genetic epidemiological technique “Mendelian randomization” (MR) [55]. In this method, genetic variants are used to proxy a traditional risk factor and estimate the causal relationship between the risk factor and the disease. Because genetic variants segregate independently of environmental confounders, the rationale is that they and the causal estimates derived from them should be less affected by confounding and other potential biases than traditional observational epidemiological studies. For example, MR studies of type-II diabetes (T2DM) have suggested that higher BMI and central fat distribution are key causes of T2DM, and that triglyceride-lowering drugs, as well as population-level interventions to reduce obesity, could help prevent T2DM [56,57,58]. Adequately powered two-sample MR studies are becoming increasingly possible as the size of GDM genome-wide association studies (GWASs) increases, enabling the investigation of causal factors underlying the risk of GDM. For example, obesity/overweight and high body mass index (BMI), conditions that are associated with both insulin resistance and inflammation [59,60,61,62,63,64,65,66,67,68], have been examined via MR. Overall, the evidence from these studies (and other designs involving, e.g., multivariable regression, paternal negative control, etc.) is robust, supporting the notion that a higher maternal BMI is causal for increased risk of GDM [69,70]. 

Another well-known risk factor for GDM is advanced maternal age at childbirth. Studies, including one with nearly a million participants, have consistently reported a progressive increase in GDM risk for mothers aged 25 years and older [45,47,71,72,73]. GDM recurrence has also been observed in nearly half of the women previously diagnosed with GDM in a Chinese cohort study (N = 10,151), with a recent and multi-ancestral meta-analysis, (N = 19,053) further suggesting that multiparous women have a higher recurrence rate of GDM compared to primiparous women (73% vs. 40%), although other factors like age and obesity might influence this relationship [47,74,75,76]. Additionally, women with a family history of diabetes are at increased risk of developing GDM, with a systematic review and meta-analysis (including 2697 women with a family history of diabetes mellitus and 29,134 women without) reporting up to 3.46 increased odds of developing GDM (95% CI: 2.80–4.27) compared to those who do not [77,78,79]. 

GDM also varies considerably across ethnicities [45,47,80,81]. For instance, a large, multiethnic, population-based study (N = 956,738) reported that South Asian women had 4.33 higher odds of developing GDM relative to Australian women [47]. Similarly, a study in the United States (N = 123,040) also reported a higher prevalence of GDM in Filipina and Asian women (10.9 and 10.2%, respectively), along with an intermediate prevalence among Hispanics (6.8%) and a lower prevalence in white Europeans and African American mothers (4.5 and 4.4%, respectively) [80]. Although this study had a large sample size, they lacked information on important risk factors, such as weight and family history of diabetes, which could influence the observed differences in prevalence across groups. Importantly, the robustness of findings for maternal age and ethnicity across many diverse studies supports them being true risk factors, although MR and other causal methods are difficult to implement for such characteristics.

## 5. Genetic Aetiology of GDM and Glycaemic Traits during Pregnancy

In addition to maternal lifestyle and environmental factors, genetics plays a role in GDM susceptibility. GWASs test for statistically robust associations between genetic variants and the trait of interest. In our review, we show how recent large-scale GWASs have provided estimates of the degree to which genetic variation influences liability to GDM and new insights into GDM disease aetiology; as mentioned previously, identified potential targets for pharmacotherapy; and, using the principles of MR, informed on potential public health preventive interventions [55,82,83].

### 5.1. Variation in Glycaemic Traits Explained by Genetics

Genetic variation is a ubiquitous contributor to individual differences in common complex traits and diseases. However, the genetic variants identified through large-scale GWASs that are robustly associated with glycaemic traits currently only explain a small proportion of the overall variance in these phenotypes. For instance, in the general population (i.e., outside of pregnancy), known genetic variants explain about 1.7% of the variance in HbA1c, 3% of FG variance, and between 4% and 14% (depending on ancestry) of the variance in 2-h glucose levels [84,85,86].

GWASs also permit the estimation of “SNP heritability” for common complex traits and diseases [87,88]. SNP heritability represents the proportion of trait variance that is tagged by SNPs on a microarray and consequently captures the sum total contribution of genome-wide significant variants and genetic variants of smaller effect scattered across the genome to overall trait heritability. It also represents that fraction of the phenotypic variance that is amenable to genetic discovery with increasing GWASs’ sample size. Recently, the SNP heritability for each of the glycaemic traits during pregnancy was estimated, with a study on East Asian women reporting estimates of approximately 5.3% for FG at weeks 16–18, 9.6% for FG at weeks 24–28, 10.2% for 1-h, and 7.8% for 2-h glucose post-OGTT at week 24–28 [83]. Interestingly, polygenic risk scores for FG during gestational weeks 24–32 in European mothers were previously reported to explain 4–7% of the variation in this trait, with studies further showing that the same FG-associated variants explained a similar proportion of variance both during and outside pregnancy [84,89,90]. Although it is interesting to see this concordance, it is also important to remember that all of these quantities (i.e., the proportion of phenotypic variance explained by a polygenic risk score, SNP heritability, and overall heritability) are population-specific ratio measures, which include environmental as well as genetic variation in the denominator and, thus, are expected to vary across different populations and circumstances/environments.

### 5.2. Robustly Associated Genetic Variants

In order to robustly identify common variants of small to moderate effect underlying common complex diseases with adequate statistical power, genome-wide association studies need to involve at least 2,000 cases and controls [91]. To the best of our knowledge, only six GWASs of GDM have been published to date [70,92,93,94,95,96], with the first study to robustly detect genome-wide significant loci for GDM being a Korean study (cases = 1,399; controls = 2,005) that identified genetic variants at the *CDKAL1* and *MTNR1B* loci that were significantly associated with risk of GDM [95].

More recently, another study attempted to gain novel insights into the genetic architecture of GDM and address limitations associated with sample size by performing a multi-ancestry GWAS meta-analysis, which included 5,485 GDM cases and 347,856 healthy controls from various population groups, including Europeans, East Asians, South Asians, Hispanics/Latinos, and Africans [70]. This research effort, led by the GENetics of Diabetes In Pregnancy (GenDIP) Consortium, identified five loci at genome-wide levels of significance—three of them novel and two of them being the known *CDKAL1* and *MTNR1B* loci [70]. Heterogeneity in estimated effect sizes across ancestries was present at two loci, *CDKAL1* and *CDKN2A-CDKN2B*, potentially reflecting differences in linkage disequilibrium (LD) patterns between ancestries (although this might also indicate that the pathophysiological mechanisms driving glycaemic dysregulation in pregnancy may vary between ancestries, which needs to be further investigated) and emphasizing the need of more studies on under-represented populations [70]. 

The largest GWAS of GDM to date was undertaken in FinnGen, a study that combined genetic data with electronic health record data to support GWASs and other genetic analyses for many health outcomes [97]. The FinnGen GWAS comprised 12,332 GDM cases (i.e., with a GDM diagnosis listed in their health record) and 131,109 parous female controls from Finland, along with an independent sample of 8,931 cases and 170,809 controls for replication (from both Finland and Estonia). The study identified thirteen genome-wide significant loci associated with GDM, with eight of the loci being novel [96]. Further analyses were also performed to determine the extent to which each locus was associated with T2DM and GDM based on their effect sizes for each condition. Loci showing GDM-predominant effects were mapped to genes linked to islet cells, central glucose homeostasis, steroidogenesis, and placental expression—these GDM-predominant effect loci include (*GCKR*, *SPC25-G6PC2*, *PCSK1*, *ESR1*, *MTNR1B*, *NEDD1*, *CMIP*, and *MAP3K15*), whereas the loci at *CDKAL1*, *TCF7L2* and *CCND2* involved T2DM predominant effects [96]. Nevertheless, the estimates of allelic effects at these loci were almost entirely in the same direction [96].

As it is possible to see in Table 2, several possible loci have been implicated in GDM [70,93,94,95,96]. However, it is important to note that while numerous genetic variants associated with GDM have been identified through GWASs, the causal genes for most loci remain unknown. Hence, this table lists candidate genes based on their proximity to the association signal, along with any additional evidence supporting their potential causality, with the caveat that these are not definitively proven causal genes but are considered candidates pending further investigation.

As glucose measurements can reflect changes in glucose metabolism across specific timepoints, and GDM is diagnosed based on an arbitrary threshold applied to these underlying quantitative traits, investigating the genetics of fasting glucose and post-OGTT glucose levels can provide deeper insights into the genetic basis of GDM. Hence, the genetic determinants of fasting and postprandial blood glucose during pregnancy have been investigated in a few studies [89,92,202,204]. One of these studies investigated 4437 mothers of different ancestries and identified five loci associated with FG (i.e., *GCKR*, *G6PC2*, *PCSK1*, *PPP1R3B*, and *MTNR1B*) [202]. Additionally, an association between 1-h glucose post-OGTT and variants in *MTNR1B* as well as 2-h glucose post-OGTT and variants in *HKDC1* has also been detected. While these associations showed important genetic determinants of glycaemic traits during pregnancy, subsequent studies have failed to replicate such associations due to limited sample sizes [89,94,204]. 

A recent study, however, explored the association between genetic variants and both GDM and several glycaemic traits (such as FG, 1-h post OGTT, and 2-h post OGTT glucose levels) in up to 26,751 East Asian mothers [92]. Although the sample size for GDM was significantly smaller than the ones from the two studies previously discussed (with solely 3317 cases and 19,565 controls), the sample sizes for the quantitative traits analysed were the largest to date, with FG measurements on 26,751 mothers at weeks 16–18 of gestation along with information on FG (N = 24,929), 1-h glucose post OGTT (N = 24,931), and 2-h glucose post OGTT (N = 24,931) values at gestational weeks 24–28. Overall, nine loci were associated with FG at gestational weeks 16–18, ten with FG at gestational weeks 24–28, seven with 1-h glucose post OGTT, and, finally, four genes associated with 2-h glucose levels post OGTT (both at gestational weeks 24–28). The genetic determinants between fasting (or baseline) glycaemic levels and glycaemic values after OGTT have been observed to be substantially different—although it is important to note that FG was measured during gestational weeks 16–18 and weeks 24–28 while 1-h and 2-h post-OGGT levels investigated were measured solely during weeks 24–28. For instance, *ABCB11*, *GCK*, *LOC101929710*, and *FOXA2* were only detected in the baseline glycaemic level analyses but not after OGTT. Additionally, *CDKAL1*, while not associated with FG at weeks 16–18, was seen to be significant in FG, 1-h glucose, and 2-h glucose post OGTT analyses at weeks 24–28 and *HKDC1*, showing a strong association with FG and 2-h glucose values post OGTT at weeks 24–28 but not with other glycaemic measurements. Further, *MTNR1B* was detected in all analyses, although the strength of association differed across timepoints, showing a stronger relationship with 1-h and 2-h post OGTT glucose values. 

**Figure 3:**
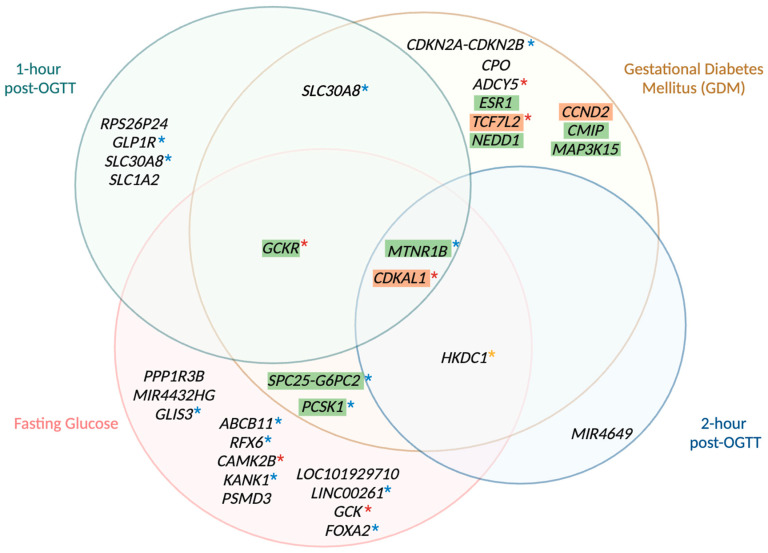
Venn diagram of genetic loci associated with gestational diabetes, 1-h glucose, fasting glucose, and 2-h glucose. This Venn diagram depicts the genetic loci that harbour variants associated with GDM, fasting glucose levels, 1-h glucose levels post OGTT, and 2-h glucose levels post OGTT, as identified by multiple genome-wide association studies of pregnant women. Each circle represents the genetic variants linked to one of the four traits. The distinct, non-overlapping areas of each circle indicate genetic variants uniquely associated with each trait, while the overlapping regions illustrate genetic variants shared between two or more traits. The central overlapping area represents variants common to all four traits. All genetic variants included in this diagram reached genome-wide significance (*p* < 5 × 10^−8^) in the studies of Kwak et al. [95], Hayes et al. [202], Pervjakova et al. [70], Elliot et al. [96], and Zhen et al. [92]. Loci with GDM-predominant effects are highlighted in green, whereas loci with type-2 diabetes mellitus predominant effects are highlighted in orange as reported by Elliot et al. [96]. Loci not highlighted remain unclassified. Loci also detected by Chen et al. [110], in the general, non-pregnant population can be distinguished based on the asterisks (*), with blue asterisks indicating associations with fasting glucose values, yellow asterisks indicating associations with 2-h post-OGTT glucose values, and red asterisks indicating associations with both fasting and 2-h post-OGTT glucose values. Colocalization analyses, however, are needed to properly compare variants inside and outside of pregnancy [269]. GDM: gestational diabetes mellitus; OGTT: oral glucose tolerance test.

Interestingly, many genetic associations with glycaemic traits during pregnancy have been detected in both non-pregnant individuals and in association with GDM (Figure 3 shows a summary of the shared and distinct genetic variants). For example, at the genome-wide level, two loci associated with 2-h glucose post-OGTT (*HKDC1* and *CDKAL1*) and twelve loci associated with FG, including *GCKR*, *G6PC2*, *PCSK1*, *MTNR1B*, *ABCB11*, *RFX6*, *CDKAL1*, *CAMK2B*, *KANK1*, *GCK*, and *FOXA2*, have been observed in both pregnant and non-pregnant populations (although proper colocalization analyses are needed to assess whether the same variants are implicated during and outside of pregnancy as this approach uses GWASs data to identify shared genetic factors across multiple related traits, helping pinpoint causal genes and mechanisms in complex diseases [269]) [66,92,95,96,110,202]. Similar trends were seen when comparing East Asian mothers in a large study on glycaemic traits in the general population (comprising up to 281,416 individuals): *ABCB11* and *FOXA2* were found in FG analyses but not in 2-h post-OGTT analyses, and *CDKAL1* was associated with both FG and 2-h glucose values [92,110]. Differences between studies included *HKDC1* being linked to both FG and 2-h glucose in pregnant East Asian mothers but only to 2-h glucose in the general population, and *MTNR1B* being significant in all glycaemic trait analyses during pregnancy but only in FG analysis in the general population [92,110]. The study of glycaemic traits in the non-pregnant population, however, only investigated FG and 2-h post-OGTT glucose values, as it is not common practice to measure 1-h glucose values post-OGTT outside of pregnancy. Hence, it is still unclear whether the genetic determinants of 1-h glucose values during and outside of pregnancy differ [110]. Although the genetic architecture of glycaemic traits during and outside pregnancy has been suggested to be shared for the most part, the genetic determinants of 2-h post OGTT glucose in pregnant women was reported to differ from the ones in the non-pregnant population [89,90]. Overall, findings suggest that although FG levels remain relatively stable outside and during gestation, the postprandial glucose levels tend to differ in order to meet the metabolic requirements imposed by fetal growth [7,12]. 

Despite recent advances in this field, the current body of research on genetic associations with GDM and glucose measurements during pregnancy remains limited, especially when compared to the extensive literature available on T2DM and glycaemic traits in the general population. Efforts to increase the genetic diversity in GWASs of GDM, especially the inclusion of underrepresented groups, are a key priority as genetic effects may vary between ancestries, a higher GDM prevalence is observed in some underrepresented groups, and the majority of the findings to date are based on European or East Asian populations [269]. Further, since identifying causal variants and the genes involved is a challenge due to the complex correlational structure of the genome, the inclusion of other ancestries where LD patterns may vary provides opportunities for improved fine mapping of genetic loci. Nevertheless, accurately addressing LD remains a challenge due to the inherent complexity of genomic structures and the need for comprehensive, high-resolution data across diverse populations.

### 5.3. Genetic Insights into the Relationship between GDM and T2DM

GWASs provide an opportunity to elucidate the degree to which diseases with different clinical presentations (e.g., T2DM and GDM) represent the same underlying disorder by examining the genetic similarity between them. This is typically performed on a locus-by-locus basis, as well as genome-wide, using methods like LD score regression, which estimates the overall genetic correlation between the traits [88]. The majority of the GWASs of GDM reported a substantial shared genetic aetiology between GDM and T2DM, with only a few genome-wide significant loci for GDM not being significantly associated with T2DM [66,92,93,94,95,96]. The largest GDM GWAS to date further used a new method called SCOUTJOY (Significant Cross-trait OUtliers and Trends in JOint York regression) to compare effect sizes of GDM-associated loci with those of T2DM. This approach evaluates if observed effect sizes across top hits conform to a uniform relationship, or whether some loci exhibit stronger associations with GDM or T2DM, while also accounting for sample overlap and estimation errors specific to each phenotype [96]. Overall, the authors reported significant heterogeneity in expected effect sizes across many of the loci, suggesting some genetic differences between the two conditions. 

Additionally, the two largest GWASs of GDM reported a genetic correlation between GDM and T2DM of around 0.70, suggesting that whilst GDM and T2DM share much of their aetiology, they may also have distinct components that contribute to their individual genetic architectures [66,96]. However, it is worth bearing in mind that these genetic correlation estimates are based on relatively small numbers of GDM cases (i.e., cases = 5485, controls = 347,856 [66]; and cases = 12,332, controls = 131,109 [96]), which also include potentially less reliable self-reported diagnoses (e.g., in the UK Biobank). This contrasts with the very large sample sizes of recent T2DM GWASs, which involve up to 428,452 cases and 2,107,149 controls [140,270,271,272]. Overall, the relatively small sample size of GDM (compared to T2DM) and the degree to which the diagnoses of GDM can be trusted limit current attempts to understand the extent to which they represent the same condition (i.e., the physiological stress of pregnancy unmasks a predisposition to T2DM that becomes diagnosed as GDM), or whether there are distinct determinants of both.

## 6. Relationship between GDM and Short- and Long-Term Adverse Health Outcomes

GDM, like many other pregnancy complications, can negatively impact both maternal and fetal health. Although a variety of short- and long-term adverse outcomes are associated with GDM, evidence is mostly based on observational epidemiological studies, and so it is unclear whether these relationships represent causality or confounding through, e.g., shared genetics. To the best of our knowledge, no studies to date have attempted to examine potential causal relationships between GDM and either maternal or fetal long-term outcomes using Mendelian randomization [55]. This is partly a consequence of the limited number of genetic variants uniquely associated with GDM (i.e., as opposed to variants associated with both GDM and T2DM) and highlights the need for increasingly large GWASs to detect such variants that could then be used in MR analyses. As such, in this section, we briefly discuss the observational association between GDM and some adverse outcomes with the caveat that these associations require validation using causal inference methods.

In the short-term, GDM (similar to T2DM and type-1 diabetes), is associated with adverse obstetric and neonatal outcomes [273,274,275,276,277,278,279,280,281,282,283,284]. A multi-ancestry meta-analysis of 14,033,990 pregnancies highlighted the increased risk of hypertensive disorders of pregnancy, induction of labour, caesarean delivery, offspring large-for-gestational-age, preterm birth, and admission to the neonatal intensive care unit in mothers with GDM [285]. Although this study was well-powered, there was substantial heterogeneity in the magnitude of association across studies, likely reflecting different methods of GDM screening/diagnosis, diverse population demographics, and methodological variations [285]. These same adverse outcomes, however, were also reported by a recent meta-analysis of 7,506,061 pregnancies, with GDM being further associated with increased odds of low one-minute Apgar score, macrosomia, respiratory distress syndrome, and neonatal jaundice [286]. Further supporting these findings, a separate study demonstrated a consistent graded linear association between both maternal fasting and post-OGTT glucose concentration and clinically relevant perinatal outcomes—such as caesarean section, induction of labour, large for gestational age, macrosomia, and shoulder dystocia—with no clear evidence of a threshold effect, a trend also seen in the HAPO study [279,287]. Interestingly, recent research has also emphasized the critical role of glucose in fetal growth, with various maternal and fetal proteins being involved in glucose homeostasis and energy metabolism and having a potential effect on offspring birth weight [288]. 

As for the long-term impacts, the majority of the studies have focused on later-life outcomes, often chronic conditions, displayed by mothers who have had a GDM diagnosis. For example, it is well-established that GDM is associated with an increased risk of T2DM, with studies showing that a previous diagnosis of GDM may carry an 8–10-fold higher risk of T2DM, and the cumulative incidence can increase markedly in the first five years after delivery [289,290,291,292,293,294,295,296]. However, as discussed previously, the discrepancy between studies on GDM and T2DM further limits the important exploration of whether they are the same or distinct conditions. Apart from T2DM, GDM has also been reported to be associated with a variety of other maternal chronic conditions, including cardiovascular diseases (CVDs) and metabolic syndrome [297,298,299,300], although causal evidence is lacking. For instance, two large meta-analyses (one including 5,390,591 women and the other 3,417,020) reported that women diagnosed with GDM may have a 2-fold higher risk of future cardiovascular events, with a 2.3-fold increased risk being observed in the first decade postpartum [297,298]. In addition, a meta-analysis containing up to 5832 women also reported that mothers with a history of GDM may have up to 4-fold increased odds of developing metabolic syndrome compared to those without [299]. Another meta-analysis (N = 13,390 participants) corroborated these findings and further reported that women may be diagnosed with metabolic syndrome as early as one year postpartum [300]. The meta-analyses of CVD and metabolic syndrome, however, all presented significant heterogeneity potentially due to varying follow-up durations; geographic biases; inconsistent definitions of GDM, CVD, and metabolic syndrome; age-related effects; potential inclusion of women with pre-existing diabetes; and a small number of studies, which together hinder precise estimation of the true risk of GDM for both CVD and metabolic syndrome [297,298,299,300].

Although the long-term impacts of GDM on offspring health have also been investigated, research in this area continues to lack long-term follow-up into adulthood, for which large trans-generational cohorts are required as well as causal analysis. The largest meta-analyses have reported that GDM (or diabetes during pregnancy in general) is associated with an increase in the child’s risk of metabolic syndrome (N = 4421) and leads to higher offspring systolic blood pressure (N = 62,344), blood glucose (N = 6423), and BMI (N = 27,311), with these associations with BMI and systolic blood pressure also being observed in a recent meta-analysis with up to 8759 participants [300,301,302,303]. In addition, studies have reported a possible association with long-term hospitalizations with diagnoses of endocrine morbidity such as diabetes mellitus and obesity in the offspring (N of the retrospective cohort study = 231,271), as well as increased odds of childhood obesity (N of the cross-sectional study = 4740)—although association with obesity was no longer significant after adjusting for maternal BMI [304,305]. Impaired glucose tolerance and future risk of T2DM has also been reported; however, the studies were extremely underpowered with sample sizes of 255 and 597, respectively [306,307]. The meta-analyses and epidemiological studies discussed also contain limitations related to variations in phenotypic measurement (e.g., the age at which offspring blood pressure was measured varied), differences in GDM definition, and inability to properly control for confounders often due to lack of information on those factors. Causal evidence is limited, and it is important to emphasize that studies on long-term health outcomes in offspring of mothers with GDM have not taken the correlation between maternal and offspring genetics into account, and these associations could be due to genetic pleiotropy [308,309,310,311,312,313].

## 7. Conclusions

GDM is a significant global health challenge, being diagnosed in approximately 14.0% of pregnancies worldwide. Its prevalence varies widely across regions and is influenced by factors such as maternal age, ancestry, obesity, and family history of diabetes. Early diagnosis and management are crucial to mitigating the adverse outcomes associated with GDM for both mothers and their offspring. However, challenges persist in understanding the underlying biological mechanisms underpinning this condition, hampering efforts to identify affected women and further improve treatment strategies. Despite recent advances and the use of GWASs to understand the genetic landscape of GDM and glycaemic traits during pregnancy, further research is needed to fully elucidate the genetic factors contributing to GDM onset and recurrence, as well as the degree to which these factors are distinct from T2DM.

## 8. Future Directions

Despite recent advances, the current body of research on genetic associations with GDM and glucose measurements during pregnancy remains limited, especially when compared to the extensive research on T2DM and glycaemic traits in the general population. To further understand the genetic influences on glucose metabolism during pregnancy, larger pregnancy cohorts and international collaborative efforts are required, with a major focus on increasing the genetic diversity within studies. Additionally, there is a growing potential for drug target MR studies as seen in the context of T2DM, which could identify medications for better treatment of GDM and test their safety, particularly as more proteomic data become available in pregnancy. By harnessing the power of large-scale studies, current limitations can be addressed, improving our understanding of GDM pathogenesis and facilitating the development of more effective diagnostic and therapeutic strategies to improve maternal and fetal outcomes.

## Figures and Tables

**Table 1:** Summary of the different diagnostic criteria for gestational diabetes mellitus. OGTT: oral glucose tolerance test. FG: fasting glucose.

				Initial Screening (Two-Step Approach)		Diagnostic Test					
Region	Organization	Year	Screening Advice	Glucose Load (g)	Cut-off	Glucose Load (g)	Fasting Glucose	1-h Glucose	2-h Glucose	3-h Glucose	HbA1c
*International*	WHO (World Health Organization)	2013	Universal	75	FG ≥ 92 mg/dL (5.1 mmol/L), or 1-h glucose ≥ 180 mg/dL (10.0 mmol/L) or2-h glucose ≥ 153 mg/dL (8.5 mmol/L)	75	≥92 mg/dL (5.1 mmol/L)	-	≥153 mg/dL (8.5 mmol/L)	-	-
	IADPSG (International Association of Diabetes and Pregnancy Study Groups)	2010	Universal	-	-	75	≥92 mg/dL (5.1 mmol/L)	≥180 mg/dL (10.0 mmol/L)	≥153 mg/dL (8.5 mmol/L)	-	-
*Americas*	ADA (American Diabetes Association)	2020	Risk-based	50	≥140 mg/dL (7.8 mmol/L)	100	≥92 mg/dL (5.1 mmol/L)	≥180 mg/dL (10.0 mmol/L)	≥153 mg/dL (8.5 mmol/L)	≥140 mg/dL (7.8 mmol/L)	-
	NDDG (National Diabetes Data Group)	1979	-	50	≥140 mg/dL (7.8 mmol/L)	100	≥105 mg/dL (5.8 mmol/L)	≥190 mg/dL (10.6 mmol/L)	≥165 mg/dL (9.2 mmol/L)	≥145 mg/dL (8.0 mmol/L)	-
	C&C (Carpenter and Coustan criteria)	1982	-	-	-	100	≥95 mg/dL (5.3 mmol/L)	≥180 mg/dL (10.0 mmol/L)	≥155 mg/dL (8.6 mmol/L)	≥140 mg/dL (7.8 mmol/L)	
	SOGC (Society of Obstetricians and Gynecologists of Canada)	2019	Universal	-	-	75	≥95 mg/dL (5.3 mmol/L)	≥190 mg/dL (10.6 mmol/L)	≥162 mg/dL (≥9.0 mmol/L)	-	-
	ACOG (American College of Obstetricians and Gynecologists)	2018	Risk-based	50	≥140 mg/dL (7.8 mmol/L)	100	≥95 mg/dL (5.3 mmol/L)	≥180 mg/dL (10.0 mmol/L)	≥155 mg/dL (8.6 mmol/L)	≥140 mg/dL (7.8 mmol/L)	-
	CDA (Canadian Diabetes Association)	2018	Universal	50	≥140 mg/dL (7.8 mmol/L)	75	≥95 mg/dL (5.3 mmol/L)	≥190 mg/dL (10.6 mmol/L)	≥162 mg/dL (≥9.0 mmol/L)	-	
	(BSD) Brazilian Society of Diabetes	2010	Universal	-	FG ≥ 85 mg/dL (4.7 mmol/L)	75	≥92 mg/dL (5.1 mmol/L)	≥180 mg/dL (10.0 mmol/L)	≥153 mg/dL (8.5 mmol/L)	-	-
*Australasia*	ADIPS (Australasian Diabetes in Pregnancy Society)	2014	Universal	-	-	75	≥ 92 mg/dL (5.1 mmol/L)	≥ 180 mg/dL (10.0 mmol/L)	≥153 mg/dL (8.5 mmol/L)	-	-
	Queensland Clinical Guideline	2015	Risk-based	-	-	75	≥92 mg/dL (5.1 mmol/L)	≥180 mg/dL (10.0 mmol/L)	≥153 mg/dL (8.5 mmol/L)	-	≥41 mmol/mol (5.95%)
	NZSSD (New Zealand Society for the Study of Diabetes)	2014	Universal	-	HbA1c ≥ 50 mmol/mol treated for GDM, HbA1c 41-49 mmol/mol required a 75g OGTT, HbA1c ≤ 40 mmol/mol required 50 g OGTT	75 and 50	≥99 mg/dL (5.5 mmol/L)	-	≥162 mg/dL (9.0 mmol/L)	-	≥50 mmol/mol (7.15%)
*Asia*	DIPSI (Diabetes in Pregnancy Study Group India)	2009	Universal	-	-	75	-	-	≥140 mg/dL (7.8 mmol/L)	-	-
	JDS (Japan Diabetes Society)	2016	Risk-based	-	-	75	≥126 mg/dL (7.0 mmol/L)	-	≥200 mg/dL (11.1 mmol/L)	-	≥48 mmol/mol (6.5%)
	JSOG (Japan Society of Obstetrics and Gynecology)	2010	Universal	-	-	75	≥100 mg/dL (5.5 mmol/L)	≥180 mg/dL (10.0 mmol/L)	≥150 mg/dL (8.3 mmol/L)	-	-
	HKCOG (Hong Kong College of Obstetricians and Gynecologists)	2016	Risk-based	-	-	75	≥126 mg/dL (7.0 mmol/L)	-	≥199.72 mg/dL (11.1 mmol/L)	-	-
	Ministry of Health (MOH) of China	2012	Risk-based	-	-	75	≥92 mg/dL (5.1 mmol/L)	≥180 mg/dL (10.0 mmol/L)	≥153 mg/dL (8.5 mmol/L)	-	-
*Europe*	EASD (European Association for the Study of Diabetes)	1991	-	-	-	75	≥108.1 mg/dL (6.0 mmol/L)	-	≥162 mg/dL (9.0 mmol/L)	-	-
	SIGN (Scottish Intercollegiate Guidelines Network)	2017	Risk-based	-	HbA1c ≥ 6.5%, or FG ≥126 mg/dL (7.0 mmol/L) or 2-h glucose ≥ 200 mg/dL (11.1 mmol/L)	75	≥92 mg/dL (5.1 mmol/L)	≥180 mg/dL (10.0 mmol/L)	≥153 mg/dL (8.5 mmol/L)	-	-
	GDA (German Diabetes Association)	2014	Risk-based	-	FG ≥ 92 mg/dL (5.1 mmol/L)	75	≥92 mg/dL (5.1 mmol/L)	≥180 mg/dL (10.0 mmol/L)	≥153 mg/dL (8.5 mmol/L)	-	-
	NICE (National Institute for Health and Care Excellence)	2015	Risk-based	-	-	75	≥101 mg/dL (5.6 mmol/L)	-	≥140 mg/dL (7.8 mmol/L)	-	-

**Table 2:** Overview of the candidate genes implicated by reported GWAS associations, describing their respective function and previously reported associations. SNP; Single-Nucleotide Polymorphism. * Associations reported in previous Genome-Wide Association Studies (GWASs) that reached the genome-wide significance threshold of 5 × 10^−8^.

Candidate Gene	Lead Associated SNP	Function of Likely Gene	Known Associations	Reported by *
*GCKR*	rs780093	The Glucokinase regulator (*GCKR*) gene encodes glucokinase regulatory protein (GKRP), an inhibitor of the glucose-metabolizing enzyme glucokinase (GCK), which regulates glucose disposal and storage [98,99,100]. GKRP also responds to increases in circulating glucose concentration by initiating a signalling cascade that results in insulin secretion and subsequent glucose uptake and storage [98,99,100].	Polymorphisms in the *GCKR* gene have been implicated in the susceptibility to T2DM and fasting glucose levels [101,102,103,104,105,106,107,108,109,110].	[96]
*SPC25-G6PC2*	rs1402837	The Spindle pole body component 25 (*SPC25*) gene encodes a mitosis-associated spindle-assembly checkpoint regulatory protein involved in kinetochore–microtubule interaction [111]. *SPC25* has been further reported to play a role in DNA repair, cell proliferation and regulation of both plasma glucose levels and β-cell function [112,113,114,115,116,117]. As for the *G6PC2* gene, it is predominantly expressed in pancreatic islets and encodes a glucose-6-phosphatase enzyme involved in the conversion of glucose-6-phosphate (G6P) to glucose and inorganic phosphate, a crucial step in glucose metabolism [117,118,119,120].	Although its role in glucose metabolism still needs to be elucidated, genetic variation in the *SPC25* gene could potentially indirectly affect cellular functions related to glucose metabolism through its involvement in cell proliferation processes. Although SPC25 does not have a clear role in glucose regulation, genetic variation in *G6PC2* has been associated with fasting and random (i.e., glucose measurements under non-standardized conditions) blood glucose levels, and HbA1c levels [107,109,110,121,122,123,124]	[96]
*CPO*	rs1597916	The carboxypeptidase O (*CPO*) gene encodes an enzyme involved in digestion of dietary proteins and peptides, assisting in the absorption of amino acids in the intestinal tract [125,126,127,128,129,130].	The exact function of *CPO* in glucose levels and diabetes needs to be further investigated; however, carboxypeptidases have been previously associated with glucose metabolism and the development of T2DM [131,132,133,134].	[92]
*ADCY5*	rs6798189	The Adenylate cyclase 5 (*ADCY5*) gene encodes an enzyme involved in the production of cyclic AMP, a key molecule involved in various cellular processes, including glucose metabolism [135,136,137,138,139].	Multiple studies have associated variants in this gene with β-cell function, fasting, and 2-h glucose levels, as well as T2DM risk [109,110,113,140,141,142,143,144,145,146,147].	[96]
*PCSK1*	rs1820176	The Proprotein Convertase Subtilisin/Kexin Type 1 (*PCSK1*) gene encodes prohormone convertase 1/3, which plays a role in the processing and activation of prohormones and precursor proteins [148,149,150,151,152,153,154]. PCSK1 was further reported to be involved in the activation and cleavage of a precursor to insulin (i.e., proinsulin) as well as in the processing of pro-opiomelanocortin (POMC), pathways that affect glucose metabolism [155,156,157,158,159,160,161,162].	Genetic association studies have implicated *PCSK1* variants in glucose homeostasis, BMI, and susceptibility to obesity [109,113,162,163,164,165,166].	[96]
*CDKAL1*	rs34499031, rs9348441, rs7766070, rs7754840	*CDKAL1* (CDK5 Regulatory Subunit-Associated Protein 1-Like 1) encodes for methyl transferase (tRNA modifying enzyme) and has been reported to influence insulin processing and secretion through proinsulin conversion, which directly affects β-cell function [167,168,169,170].	Studies have implicated *CDKAL1* variants in T2DM risk and abnormal β-cell function [104,109,110,171,172,173,174].	[70,92,95,96]
*ESR1*	rs537224022	The Estrogen Receptor 1 (*ESR1*) gene plays a crucial role in insulin sensitivity and glucose metabolism through its contributions to glucose uptake and utilization [175,176,177,178,179,180].	Genetic association studies have reported variants in *ESR1* to be associated with T2DM and fasting plasma glucose [181,182,183,184,185].	[96]
*SLC30A8*	*rs13266634*	*SLC30A8* (solute carrier family 30, member 8) gene encodes an islet zinc transporter (ZnT8), involved in zinc transport into β-cell insulin-secretory granules and essential for insulin packaging and secretion [186,187,188,189].	Studies have reported associations between *SLC30A8* genetic variants and plasma glucose levels, and susceptibility to T2DM [104,106,110,113,190,191,192].	[92]
*CDKN2A-CDKN2B*	rs1333051, rs7019437, rs10811662	*CDKN2A* and *CDKN2B* (Cyclin-Dependent Kinase Inhibitor 2A and 2B, respectively) are genes controlling cellular proliferation through their role in cell cycle regulation [193,194,195].	Variants in the *CDKN2A* and *CDKN2B* genes have been associated with susceptibility to T2DM, β-cell proliferation, and glucose levels [109,110,145,196,197,198].	[70,96]
*HKDC1*	rs9663238	The hexokinase domain-containing 1 (*HKDC1*) gene encodes a hexokinase protein and plays a crucial role in the regulation of glucose homeostasis through its effect on whole-body glucose disposal and insulin sensitivity [199,200].	Genetic association studies have reported that variants in *HKDC1* are associated with glucose homeostasis, HbA1c, plasma glucose levels in non-pregnant individuals, and liver enzyme alanine aminotransferase levels [84,110,201,202,203,204,205,206].	[70]
*TCF7L2*	rs34872471, rs7903146	The *TCF7L2* (Transcription Factor 7-Like 2) gene encodes a transcription factor involved in cellular signalling pathways and glucose metabolism, playing an important role in the synthesis and maturation of proinsulin, as well as β-cell proliferation [207,208,209].	Variants in *TCF7L2* have been implicated in insulin secretion and resistance, plasma glucose levels, β-cell function, and susceptibility to T2DM [109,110,140,198,210,211,212,213,214,215,216,217,218].	[70,96]
*MTNR1B*	rs10830963, rs10830962	The Melatonin Receptor 1B (*MTNR1B*) gene encodes melatonin receptors, which are involved in modulating insulin secretion and glucose metabolism in pancreatic β-cells [219].	Variants in the *MTNR1B* gene are associated with insulin response, plasma glucose levels, risk of T2DM, and offspring birthweight [109,110,112,141,220,221,222,223,224,225,226,227,228,229,230,231,232,233].	[70,92,95,96]
*NEDD1*	rs74628648	The *NEDD1* (Neural precursor cell expressed, developmentally down-regulated gene 1) gene encodes a protein that interacts with γ-tubulin and forms a complex that is targeted to the centrosome for spindle assembly and centriole duplication; hence, it is involved in mitosis [234,235,236,237].	Although the role of *NEDD1* in diabetes still needs to be elucidated, it is speculated that the protein encoded by this gene might be linked to glucose metabolism through its involvement in cellular division which could, for instance, affect β-cells.	[96]
*CCND2*	rs76895963	The *CCND2* (Cyclin D2) gene encodes a protein involved in cell cycle regulation and proliferation, being associated with β-cell replication and expansion, which consequently affects beta-cell mass and function [238,239,240,241,242,243,244,245,246,247].	It is hypothesized that Cyclin D2 may indirectly affect insulin secretion through its role in regulating β-cell proliferation and function, with studies also reporting an association between variants in *CCND2* and both blood glucose levels and T2DM [109,110,248].	[96]
*CMIP*	rs2926003	Encodes a c-Maf-inducing protein involved in multiple signalling pathways and associated with inflammatory responses [249,250,251].	Variants in *CMIP* have been reported to be associated with T2DM, obesity, and obesity-related traits [252,253,254,255,256,257,258,259,260].	[96]
*MAP3K15*	rs56381411	The *MAP3K15* (Mitogen-Activated Protein Kinase Kinase Kinase 15) gene encodes a protein kinase that regulates apoptotic-mediated cell death and stress response [261,262,263,264,265].	Other genetic variants within this gene have previously been associated with risk of T2DM, blood glucose, and glycosylated haemoglobin (HbA1c) levels possibly due to its involvement in pancreatic islet cell or stress response pathways [140,266,267,268].	[96]

## Data Availability

Not applicable.
